# Research of the Ball Burnishing Impact over Cold-Rolled Sheets of AISI 304 Steel Fatigue Life Considering Their Anisotropy

**DOI:** 10.3390/ma16103684

**Published:** 2023-05-11

**Authors:** Stoyan Slavov, Diyan Dimitrov, Mariya Konsulova-Bakalova, Lyubomir Si Bao Van

**Affiliations:** 1 Department of Mechanical Engineering and Machine Tools, Technical University of Varna, 9010 Varna, Bulgaria; mbakalova@tu-varna.bg (M.K.-B.); lubomir.van@tu-varna.bg (L.S.B.V.); 2 Department of Mechanics and Machine Elements, Technical University of Varna, 9010 Varna, Bulgaria; dm_dimitrov@tu-varna.bg

**Keywords:** surface regular reliefs, ball burnishing, optimal toolpath generation, fatigue life, austenitic stainless steel, Bayesian rule, cold-rolled sheets

## Abstract

The present work focusses on the research of the plastic deformation accumulated effect obtained after two different plastic deformation treatments, over the fatigue life of AISI 304 austenitic stainless steel. The research is focused on ball burnishing as a finishing process to form specific, so-called “regular micro-reliefs” (RMRs) on a pre-rolled stainless-steel sheet. RMRs are formed using a CNC (Computerized Numerically Controlled) milling machine and toolpaths with the shortest unfolded length, generated by an improved algorithm, based on the Euclidean Distance calculation. The effect of the predominant tool trajectory direction during the ball burnishing process (which can be coinciding or transverse with the rolling direction), the magnitude of applied deforming force, and feed-rate is subjected to evaluation using Bayesian rule analyses of experimentally obtained results for the fatigue life of AISI 304 steel. The obtained results give us reason to conclude that the fatigue life of researched steel is increased when directions of pre-rolled plastic deformation and the tool movement during ball burnishing are coincident. It also been found that the magnitude of deforming force has a stronger impact over the fatigue life, than the feed-rate of the ball tool.

## 1. Introduction

Austenitic stainless-steel grades that fall within group 300 have wide application as material due to their higher level of corrosion resistance, good plastic deformation behavior, and machinability, even in cold state. Usually, they can be found on the market as cold- or hot-rolled rods, tubes, or sheets. 

The SS 304 can be used directly to produce the final products in the nuclear power, space, petrochemical, food, pharmaceutical industries, etc. With this steel a high degree of strain hardening can be achieved that results in particularly good mechanical properties after deformation [1,2]. However, despite its good behavior in plastic deformation, various defects can occur with excessive hardening. They can be attributed to the rolling process more than to the steel grade itself. Different flaws occur in the rolled material such as, for example [3,4,5,6], surface inconsistence, non-uniform deformation, wavy or barrel edges, internal pores, non-metallic inclusions, alligator type or hydrogen cracks, etc. Roll stiffness, dimensional tolerances, geometric shape deviation, rolling mill geometric errors, and improper lubrication during the rolling process are in the basis of the above-mentioned defects. Elastic roll deformation may lead to irregular sheet thickness across the material but also to waviness in the edges of the material and cracks at its center because it is subjected to tension while the edges are subjected to compression at the same time. 

Some authors [7,8] studied the effects of prestraining and different manufacturing processes on the fatigue properties of rolled specimens. Chang-Yeol Jeong et al. reported a fatigue limit higher than the monolithic yield strength (YS) of low carbon steel sheets under pulsating cycles (R = 0) due to cycling strain hardening. The prestraining increases YS, which leads to improved fatigue performance of the material. Regarding the tensile properties after predeformation, the yield strength increases with a higher amount than the fatigue limit does. At the same time, Fredriksson et al. concluded that the fatigue life is not very sensitive to prestraining with the specimens being DP400, DP600, and HSLA500 steels. 

For final mechanical properties when rolled specimens are used, the residual stress should also be considered. The main factors affecting the residual stress are the role’s size, the thickness of the sheet, the system’s lubrication, and the specimen’s total reduction. Other authors [9,10,11] also have studied the influence of these factors. It is experimentally proven that reduction, achieved by unlubricated roles, leads to increased residual stress in aluminum and brass strips. With low friction, the distribution of the residual stress depends on the diameter of the role and the decrease in the thickness. By reducing the diameter of the rollers or the total reduction, the residual stress is also decreased. 

It is known that 304 belongs to the metastable stainless-steel group (i.e., group 300), which means that some martensitic transformation can occur when it is subjected to certain plastic straining [12,13,14]. 

In [12] the authors are using the specimens prepared by cold rolling of AISI 301 stainless steel with a 48% reduction leading to the formation of a high amount of strain-induced martensite. The Spherical Harmonic Analysis method is used to study the grain-orientation-dependent residual stress in cold-rolled stainless steel, prior to and after annealing. A correlation between the residual stress within a grain in the cold-rolled samples and the orientation of the grain was found. For some of the specimen directions, the local residual stresses within a grain vary widely, from tension to compression, and its magnitudes could be much larger than the phase stress. Primarily, annealing leads to a re-distribution of the residual stress and it reduces the orientation anisotropy of residual stress in the material. The results show that annealing can reduce residual stress after rolling. 

Another suitable way to improve the material structure, but in the material surface layer, is using finishing methods such as sandblasting, shot peening, or different approaches for ball burnishing (BB). There are lot of reported results of the positive effects and enhancements of the integrity for austenitic stainless steels’ surface layer, after the application of different finishing methods based on plastic deformation. For example, Chui et al. [15] report that after applying a fast multiple-rotation rolling treatment, microhardness of the test specimens, made of 316L steel in the top surface layer, has a significant increase, due to refinement of grains, deformation-induced α-martensitic transformation, and work-hardening in the specimen’s surface layer. In Jerez-Mesa et al. [16], the effects of both ultrasonic vibration-assisted and non-vibration BB approaches are investigated over the subsurface microstructure of a transformation-induced plasticity AISI 301LN alloy. As a result, the authors report that the pre-existing microstructure of the material has a strong correlation with the effect of BB treatment. Nagîț et al. [17,18] have investigated the effects over the physical–mechanical properties, the microgeometry, and some tribological characteristics of different surface textures, obtained after different finishing processes. They conclude that the best results are obtained after applying a ball vibroburnishing process. In Attabi et al. [19] both the surface topography and microhardness of 316L after applying BB are studied. The authors confirm that the multi-pass BB process led to significant improvement of the surface roughness and microhardness in the surface layer. Dzierwa and Markopoulos [20], who also have studied the surface roughness and residual stresses of the 42CrMo4 steel, reached similar conclusions about the positive effects of the BB process. A large amount of published works [21,22,23,24,25,26,27,28,29,30] show that the application of different finishing methods, related to plastic deformation in the surface layer of the different materials, can significantly increase the surface integrity and fatigue life of the parts, subjected to the cyclic load.

Sometimes, in addition to the increase in the material’s surface layer integrity, particular functional properties of the parts’ contact surfaces must be obtained. Specific surface textures, which are called “regular micro-reliefs” (RMRs) can be formed on the parts’ surfaces, using a modification of the classical BB process, called “vibratory ball burnishing” (VBB) [31,32]. The main difference between the two methods is the more complex toolpath that performs the ball tool in the VBB process, which is near to a sinusoidal shape. Resulting RMR textures after VBB applying are classified into five diverse types, depending on the way the plastic deformation traces from the ball tool superimposes each other [31]. These RMRs can assure additional enhancements of the BB-processed surfaces’ operational characteristics, such as reliably retaining lubricants on contact surfaces, removing the wear remnant particles, scattering the reflected radiations [33], etc. Of particular interest is the RMR from the IV-th type, as it completely covers the burnished surface, and consists of regularly arranged rectangular or hexagonal cells.

**Figure 1:**
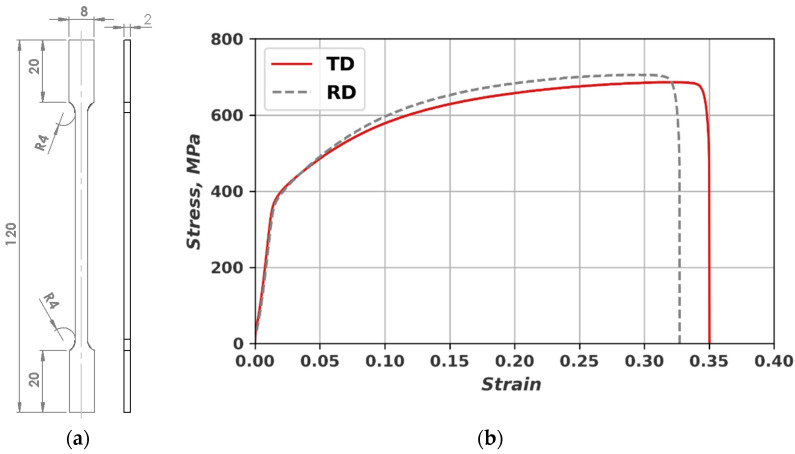
(**a**) Dimensions of the tensile specimens; (**b**) Stress–strain curves: 1—tensile specimen cut in RD, 2—tensile specimen cut in TD.

The VBB process was initially performed using manually operated machine tools, equipped with external vibration-generating devices that make the ball tool oscillate around the main trajectory direction [31]. In this way, the complex toolpath needed (see Figure 1) to obtain all five types of RMRs can be achieved. Nowadays, similar complex toolpaths can be mathematically calculated by using CAD-CAM and appropriate algorithms [34,35]. Then, employing the ability of the contemporary CNC-machine tools to interpolate two (or more) driven axes, they can be performed without needing to use any enforced oscillations of the ball tool [36]. However, the increased length of the toolpaths when processing RMRs by using BB is considered as one of the drawbacks from an operation performance perspective. Therefore, research of suitable approaches for optimization of the unfolded toolpath length in BB is also a subject of interest.

However, formation of the RMRs using BB processing causes an additional plastic deformation in the surface layer of the material that already is plastically deformed to a certain degree due to pre-rolling. Depending on the predominant direction of the ball tool movement during the BB process, the direction of that additionally induced plastic deformation can coincide with the rolling direction (RD) or can be transversal to it (TD). Wibowo et al. [37] have studied deformation-induced martensite in 316L stainless steel through tensile pre-strain deformation in the RD and TD at various % pre-strain. They found that the induced volume fraction of martensite is greater in RD, than in TD under the same pre-strain tensile conditions. This gives us reason to assume that the mechanism induced by BB plastic deformation in the surface layer of grades 300 austenitic steels could be quite similar in the case of RMR formation. This means that the predominant direction of the ball tool movement could have a distinguishing impact over some operational properties (such as the fatigue life for example) of cold-rolled sheets that are made of such steel grades. If the deforming force in the BB process has a greater pre-set magnitude, this could exceed the ultimate strength of the burnished material in the surface layer and lead to the deterioration of its physical and mechanical properties. 

Therefore, the first task in the present work is related to improving the previously used algorithm for the generation of the BB-operation toolpath to minimize its unfolded length. The second one is focused on the impact of the resulting mechanical properties’ anisotropy over the fatigue life of specimens made of AISI 304 austenitic stainless steel after formation RMR from the IV-th type by BB. In current research, the Bayesian approach is used to evaluate the contribution of the BB process over the material’s fatigue life. The results are expressed by the gain of the fatigue life, as result of applying BB process, instead by the absolute number of cycles until fatigue failure of the material. In this way, the impact of some test condition factors such as slight differences and/or imperfections of the prepared test specimens, small fluctuations in the fatigue test regime parameters, etc., are avoided.

## 2. Materials and Methods

### 2.1. Material

A cold-rolled sheet of austenitic stainless-steel grade AISI 304 with 2 mm thickness was used. The particular chemical composition of the used material is measured in our lab using a Bruker Corp., USA, model S1 Sorter XRF analyzer. The resulting chemical composition in wt% is 0.022% C, 18.44% Cr, 8.35% Ni, 0.12% Mo, 1.87% Mn, 0.31% Cu, and the rest is Fe. 

The mechanical properties of the AISI 304 steel were determined experimentally by using a universal testing machine (FU1000) and the Vickers hardness tester. The tensile test is carried out using flat specimens (see Figure 1a), which were cut from the SS 304 sheet parallel to the rolling direction Usually, specimens cut in a 45° angle are also considered but, since the fatigue testing is conducted only in RD and TD the stress–strain curves for only for those directions are shown in Figure 1b.

The obtained results for the mechanical properties of the test sheet material are given in Table 1. The results are summarized from testing 3 specimens from both groups. Mean value for hardness and uncertainty are calculated based on 10 measurements. 

As can be seen from Table 1, the YSs are similar; slightly higher UTS and lower elongation of RD-cut specimens testified that it is likely to have anisotropy in the mechanical properties, due to the manufacturing process.

### 2.2. Fatigue Failure Test Specimens and Setup

**Figure 2:**
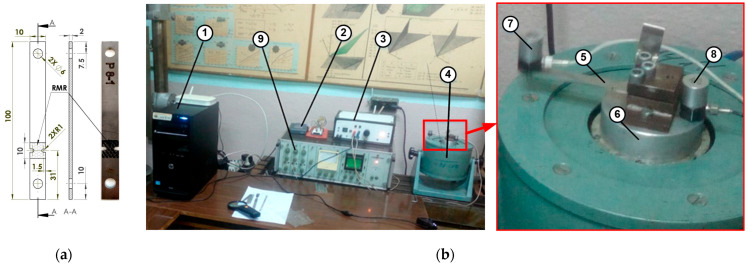
(**a**) Shape and dimensions of the fatigue test specimens; (**b**) Fatigue test setup, based on reversal bending approach.

The fatigue specimen’s shape and dimensions, used in the current experimental study, are shown in Figure 2a. Its design is pursuant to the fatigue test setup that works at resonance frequency exiting fundamental bending mode and, thus, realizing reversal bending fatigue load, shown in Figure 2b. The setup can be approximated with a single degree of freedom model of the cantilever beam with a tip mass, in Table 2. The length of the specimen is chosen from the natural frequency condition.

Taking the length of the cantilever model *L =* 75 mm gives the value *f*_1_ = 80 Hz. This frequency is experimentally obtained as a limitation of the vibration shaker used. Normal stress in the beam is proportional to its curvature; therefore, the maximal value of the normal stress in the narrow section (*b_c_ =* 5 mm), positioned at length *x_c_* from the clamped end of the beam, is:

(1) $${\max\sigma }_{xx}=Ec\cdot \frac{I}{I_{c}}\cdot {\tilde{y}}^{\prime \prime }\left(x_{c}\right)\cdot \left|y\left(L,t\right)\right|,\;$$

where $c=\frac{h}{2}\;;I_{c}=\frac{b_{c}h^{3}}{12}\;;\;\left|y\left(L,t\right)\right|=\frac{\left|\ddot{y}\right|}{{\left(2\pi f_{1}\right)}^{2}}$—displacement amplitude at the tip.

As can be seen from (1), the fatigue test is strain controlled. Stress amplitude is calculated using the tip acceleration. The real specimen is designed with a length of 100 mm, and a concentrator positioned at 31 mm from the end, considering the clamped length and the position of the central vertical axis of the tip-mounted accelerometer. To calculate the critical stress at the edges stress concentrator factor *K*_1_ = 1.3 [39] should be used for the chosen type of concentrator. The final design is proved using the FEA of a 3D model. Details for the FE solution are given in our previous research papers [27,28]. 

The fatigue test setup (see Figure 2b) consists of the personal computer (pos. 1) which controls the MyRio 1900 (National Instruments, Austin, TX, USA) device (pos. 2), for automatic adjustment of the excitation signal frequency. The excitation signal is amplified by the linear power amplifier (pos. 3) and drives the shaker (pos. 4). The fatigue test specimen (pos. 5) (see Figure 2a) is clamped onto the shaker (pos. 6), and harmonically excited with a frequency close to its fundamental bending mode shape. Two accelerations, one at the tip of the test specimen and the second at the shaker table, close to the clamped end, are measured using two piezo-ceramic accelerometers KD-35 (pos. 7 and 8). The signal from the accelerometers is also amplified, using the amplifiers (pos. 9), and via the MyRio 1900 device it enters back for processing to the personal computer (pos. 1). To keep constant amplitude stress during every fatigue test, a LabView-based routine is developed, implementing a phase-locking loop which dynamically controls the amplitude and the frequency of the excitation signal. Thus, all the specimens are tested in the same loading conditions. The shaker is dynamically controlled to follow the resonant frequency in the range 79–82 Hz, in order maintained a constant amplitude stress of 310 MPa in the concentrator area of the tested specimens (see Figure 2a). The test is interrupted when the resonance condition cannot be kept. 

### 2.3. Design of the Experimental Research 

The results from conducted experimental research [27,28] shows that among the regime parameters of the BB process, the deforming force *F*, *N*, and feed-rate *f*, mm/min, have the highest impact over the material surface layer hardening and fatigue strength of the 304 austenitic stainless steel. The rest of BB process regime parameters, such as amplitude *e*, mm, and number of the wavelength *i*, of the complex toolpath, along with the diameter of the ball tool *d_b_*, mm, and type of the used lubricant have insignificant effect. Only the deforming force and feed-rate are chosen, according to full factorial orthogonal experimental design that consist of two factors, which are varied on three levels [40]. The full experimental design with all combinations of coded and natural values of the two participating regime parameters is shown in Table 3. 

### 2.4. Description of the RMR Formation by BB Process 

#### 2.4.1. An Improved Algorithm for BB-Operation Toolpath Generation

It is known that the toolpaths of the ball tool in vibratory BB must have a near-sinusoidal shape, in order for RMRs to be formed on the processed surfaces [41]. This leads to an increase in the unfolded length of the toolpath in comparison with conventional BB operations. Some examples of such mathematically calculated toolpaths, used in BB operations, on the CNC machines are given in [28,33]. The task to optimize the BB process carried out using CNC equipment to minimize the unfolded toolpath length is relevant in the context of the contemporary requirements of production processes. In this regard, in the current work is an improved approach for connecting the calculated points from the BB toolpath that is proposed, as an attempt to optimize its unfolded length. 

**Figure 3:**
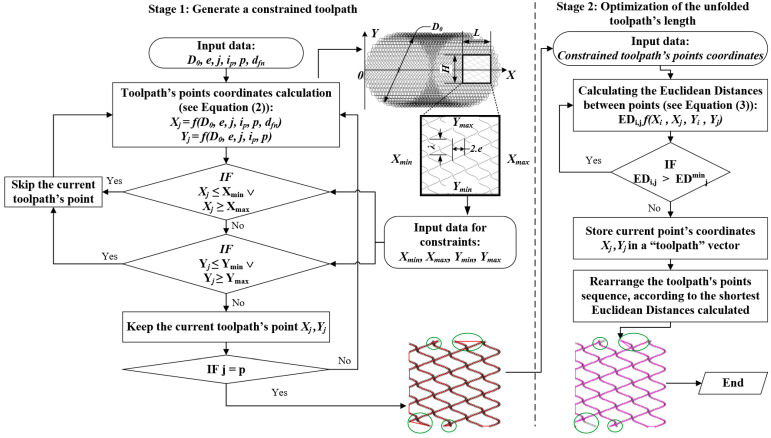
An algorithm for obtaining optimized toolpath for BB operation, based on Euclidian Distance calculation. Green circles denote the areas with optimized toolpath’s length.

**Figure 4:**
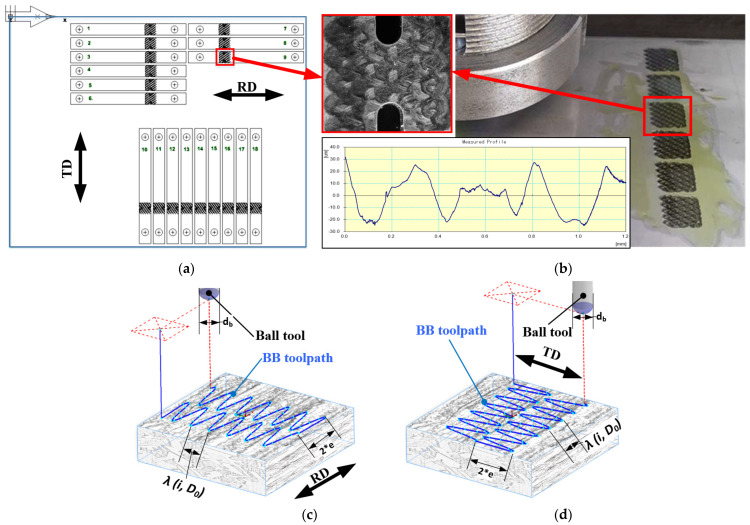
(**a**) Arrangement of the experimental specimens within the SS 304 sheet; (**b**) Topography image (zoomed in the red box) and profile measured for the RMR from IV-th type formed by BB operation; Schematic diagrams of the deforming element predominant reciprocating movement direction: (**c**) RD means along to the rolling direction; (**d**) TD means transverse to the rolling direction.

The coordinates of the points from the unconstrained toolpath, which deploys in the X–Y coordinate plane, are calculated using the following pair of equations (Figure 3) [34]:

(2) $$\left|\begin{array}{c} X_{j}=\left(e\cdot \cos\left(\frac{2\cdot \pi \cdot j}{p}\right)+\frac{1}{2}\cdot \sqrt{D_{0}{}^{2}+4\cdot e^{2}\cdot \cos{\left(\frac{2\cdot \pi \cdot j}{p}\right)}^{2}}\right)\cdot \sin\left(\frac{2\cdot \pi \cdot j}{p}{+i}_{p}\cdot j\right){+d}_{\mathrm{fn}}\cdot j\\ Y_{j}=\left(e\cdot \cos\left(\frac{2\cdot \pi \cdot j}{p}\right)+\frac{1}{2}\cdot \sqrt{D_{0}{}^{2}+4\cdot e^{2}\cdot \cos{\left(\frac{2\cdot \pi \cdot j}{p}\right)}^{2}}\right)\cdot \cos\left(\frac{2\cdot \pi \cdot j}{p}{+i}_{p}\cdot j\right) \end{array}\right.$$

where p is the number of the toolpath points; j is the index of the current point from the toolpath (j = 0, 1, 2,… p); D_0_, mm, is the toolpath’ diameter; e, mm, is half of the amplitude of the sinewave; d_fn_, mm, is the linear displacement along the X-axis for one rotation 2·π; and i_p_ is the fractional part of the ratio i = π·D_0_/λ, where λ is the length of the sinewave (Figure 4c,d).

After that, the toolpath is restricted within the boundaries of the burnished planar surface that in the current case has a square shape and the dimensions 10 × 10 mm. If a direct stich approach is used the adjacent toolpath’s segments, which fall within the boundaries, have connected each other with straight lines as can be seen from Figure 3.

However, this often results in elongation of the toolpath’s unfolded length, which is more perceptibly near to the boundaries of the burnished surface’s zone. To optimize the overall length of the toolpath, an algorithm based on the calculation of the Euclidean Distance [42] (i.e., the shortest possible distance between two points in the X–Y plane) between the toolpath’s points is proposed (see Figure 4). The algorithm uses the following logical condition to calculate the Euclidean Distances between toolpath’s points, which falls in the burnished section area:

(3) $$ED_{i,j}=\left\{\begin{array}{c} \;\infty ,\;i=j\\ \sqrt{{\left(X_{i}-X_{j}\right)}^{2}+{\left(Y_{i}-Y_{j}\right)}^{2}},\;i\;\neq j \end{array}\right.,\;$$

where *X_i_*, *X_j_* and *Y_i_*, *Y_j_* are the toolpath’s point coordinates in the X–Y plane; *i* is the row; and *j* is the column indexes of the toolpath’s points. 

After the square matrix ED is filled with all of the calculated Euclidian Distances between the toolpath’s points in the current row, the algorithm finds the smallest value obtained. In the next step it compares the smallest value to all other calculated distances in the current row of the square matrix ED, looking for a match. Where a match is found, the current point sequence index from the constrained toolpath is retrieved in the “toolpath” vector. Then, they are rearranged according to their smallest EDs calculated. In this way, the optimized sequence of points is output in the resulting matrix, which consists of their coordinates *X_j_, Y_j_* and describes the shortest toolpath within the BB area boundaries. Comparing the toolpaths obtained after stage 1 and 2 of the algorithms, shown in Figure 3, the shortening effect in the toolpath is observed near the upper and lower boundaries of the square BB area. At the same time, the toolpath remains unchanged inside to the burnished zone, which guarantees obtaining RMRs with desired characteristics of the surface topography patterns. The toolpath optimization is not expected to significantly affect the resulting RMR or the material fatigue characteristics. The main expected effect is to shorten the length of the tool path, thus shortening the cycle time of the BB processing.

#### 2.4.2. Description of the BB-Operations Parameters

Processing of the test specimens using BB is performed using a CNC-milling machine HAAS, TM-1, equipped with a specially designed ball burnishing tool [43] that can provide a deforming force up to 3200 N and has an integrated force sensor for the precise adjustment of the deforming force. As a lubricant, Mobil DTE 25 hydraulic oil is used. Completely regular reliefs from the IV-th type are formed on both sides of the AISI 304 steel sheet, with topography as shown in Figure 4b. The profile of the obtained RMRs were measured five-fold using a roughness tester Mitutoyo, America Corporation, USA, model SurfTest SJ-301 and the averaged values obtained according to the height’s criteria (see ISO 4287) are Ra = 5.25 μm and Rz = 31.21 μm. 

In the current research, the auxiliary regime parameters of the BB process are fixed at their optimal values in order to obtain RMR from the IV-th type. They are as follows: *e =* 1 mm, *i =* 600.15, and *d_b_ =* 14 mm. Considering the values for the parameters *e* and *i,* the sinewave parameters of the ball toolpath will have the resulting values, as follows (see Figure 4c,d):

- The amplitude *е* = 1 mm,
- The length λ(I, D_0_) = π·D_0_/i ≈ 2.09 mm (if D_0_ is set to 400 mm).

Set in that way, the RMRs cells are expected to have a near-to-square shape, and size of approximately 2 × 2 mm (see Figure 4b) within the boundaries of the square burnished area with dimensions of 10 × 10 mm. 

After formation of all areas with RMR on the AISI 304 sheet surface according to the experimental design (see Table 3), the test specimens are cut off from the sheet, using the fiber laser cutter machine GN NCF 3015. To eliminate the scratches and residual stresses and to ensure the low fillet radius, the side area in the zone of the stress concentrator is polished electro-chemically, prior to fatigue testing. 

### 2.5. Metodology of Data Analyses Based on Bayesen Rule

Usually, fatigue life is modeled as a Weibull or Log-Normal distribution. So, if the Log-Normal distribution is chosen, the problem is to estimate the posterior distribution of the parameters of the Normal distribution in logarithmic scale. Knowing the data generation model, its parameters can be inferred using the Bayesian approach. This approach gives not only the point estimations but also distributions from which the credible interval for parameter values can be estimated.

(4) $$f\left(\theta |D\right)=\frac{f\left(D|\theta \right)\times f\left(\theta \right)}{f\left(D\right)}$$

In the Bayes Formula (4), $f\left(D|\theta \right)$ is a likelihood function expressing the probability of generating data *D* from the certain probability density function with parameters *θ;* $f\left(D\right)$ is the marginal data probability called “Evidence”; and $f\left(\theta \right)$ and $f\left(\theta |D\right)$ are prior and posterior (conditional to data) distributions of the unknown *θ* parameters. To find the posterior probability distribution $f\left(\theta |D\right)$, prior knowledge $f\left(\theta \right)$ should be incorporated. Prior distributions can be broad “weekly informative” or flat “uninformative” such that posterior distributions can be inferred mostly from the data. The denominator in (4) $f\left(D\right)$ is just a normalizing constant and usually is omitted. 

This problem has a closed form solution if the proper conjugate prior distributions (Normal, Inverse Gamma) are chosen. 

On the other side, it is known that experimental fatigue data is characterized by high variance and the probability of outliers is relatively high. Sources of uncertainty are specimen preparation, material inhomogeneity, and testing conditions. Outliers mainly affect the mean value estimation. To decrease the influence of outliers it is better to model the fatigue life using Student’s *t* than Normal distribution. *t*-distribution has the 3rd parameter degrees of freedom (ν) which control the tails. For example, the 95% of the density of Normal distribution is in range ± 2σ; similar values have the *t*-distribution with 30 or more degrees of freedom (ν ≥ 30), but if ν = 1 the 95% of probability density is in range ± 12.7σ.

The probabilistic model with non-conjugate distribution can be empirically sampled using the Markov Chain Monte Carlo (MCMC) algorithms, available in all the software packages for Bayesian statistics. 

As the posterior distributions of the parameters are obtained, the predictions and new data can be inferred from the posterior predictive distribution (PPD) (5) which incorporates uncertainty of the data and uncertainty of the inferred model parameters.

(5) $$f\left(D^{*}|D\right)=\int_{\theta }^{}f\left(D^{*}|\theta \right)f\left(\theta |D\right)d\theta \;$$

In (5), D* is newly generated data conditional to experimental data *D*. Proper PPD will have similar statistical properties to real experimental data and can be interpreted as the possible outcomes of experiment replications. 

## 3. Results

### 3.1. Fatigue Life of Non-BB (Raw) Specimens

Specimens with dimensions given in Figure 2a without BB were selected from rolled sheet material. Some of them were chosen in the rolling direction (RD) and some of them in the transverse direction (TD). After the fatigue test, the following results for the fatigue life, expressed in cycles to failure (*N_f_*), are obtained (Table 4). Cycles to failure *N_f_* are converted to log scale and normalized by extracting the mean and dividing to the standard deviation of log values. As it can be seen in Table 4, the fatigue life of specimens cut in RD is in range (4.94–6.71) × 10^4^ cycles, higher than the TD specimens where the fatigue life is in range (3.93–4.73) × 10^4^ cycles. This is a reasonable dispersion of fatigue life. Results can be influenced by several sources of variability, including specimen geometry (especially for the rectangular section specimens, edges should be with equal filets; sharp edges, or fillets with a bigger radius can significantly affect the fatigue life), material variability (austenitic steels are metastable; strain-induced martensite transformation is strongly affected by chemical composition), loading variability, environmental effects etc.

Since the specimen size of both groups is relatively low and the values are close, it is reasonable to test the Null-hypothesis for the difference between the means of the two groups. It is achieved using Bayesian estimation according to Kruschke’s suggestion [44]. Each group is presented as a *t*-distribution with unknown parameters (mean, standard deviation, and degrees of freedom). For unknown parameters, weekly informative prior distributions are chosen. The model is sampled using the MCMC algorithm. Two chains with 11,000 samples with burn in the first 1000 are generated. From the posterior distribution the samples’ deterministic variables called “Difference Of Means” and “Effect Size” are obtained. If 95%HDI of the empirical distribution of this variable excludes the region of practical equivalence (ROPE) around zero (±0.05) it can be rejected with a 95% level of confidence. The values of the ROPEs are chosen according to recommendations given in [45]. Mean values of prior distributions (*m_i_*) are taken from the empirical mean of the data.

(6) $$\mathrm{Likelihood}\;\mathrm{Log}(N_{f}){,}_{i}\;\sim \;t\left(\mu_{i},\sigma_{i},\nu \right),\;i\in RD,\;TD\;\mathrm{Prior distributions}\;\mu_{i}\;\sim \;N\left(m_{i},s\right)\;,\;i\in RD,\;TD\;\sigma_{i}\;\sim \;\mathrm{Uniform}\left(0,\;3\right),\;i\in RD,\;TD\;\nu \;\sim \;\mathrm{Exp}\left(\lambda \right)+1\;m_{i}=\left(\;0.5\;,-1.0\right),\;\in RD,\;TD\;,\;\;s=3,\;\;\lambda =\frac{1}{29}\;\mathrm{Deterministic variable}$$

$$\mathrm{Diference Of Means}=\mu_{TD}-\mu_{RD}$$

$$\mathrm{Effect Size}=\frac{\mu_{TD}-\mu_{RD}}{\sqrt{\frac{\left(\sigma_{TD}^{2}+\sigma_{RD}^{2}\right)}{2}}}$$

**Figure 5:**
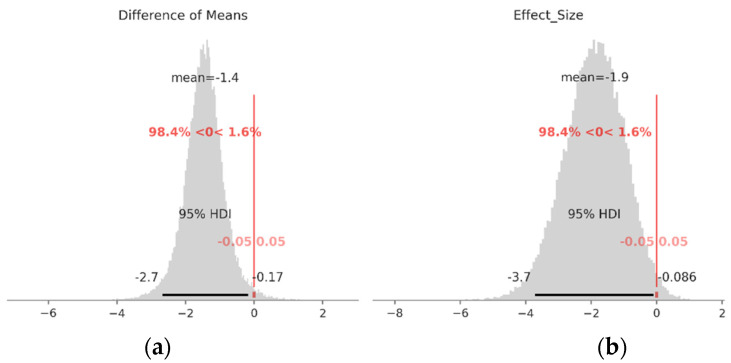
Posterior distributions of determinant variables (Supplementary S1): (**a**) Difference of means; (**b**) Effect size.

A summary of the results is given in Table 5. Posterior distributions of the means of both groups (*µ****_RD_****, µ****_TD_***) have mean values close to the empirical mean of the experimental data. Posterior distributions of standard deviations ($\sigma_{RD},\;\sigma_{TD}$) of both groups have similar locations, but the scale (sd) is higher for the group of TD specimens. Posterior distribution of the degrees of freedom (***υ***) variable has a mean of about 32, which is an indicator for the normality of the data. Posterior distributions of determinant variables with sample mean 95%HDI and ROPE visualizations are shown on Figure 5. As can be seen, the 95%HDI of the variables “Difference of means” and “Effect Size” are out of the ±0.05 ROPE, so H0 is rejected. This means that specimens cut in the RD exhibit a longer fatigue life than those cut in the TD. As was expected, materials that are cut in the RD exhibit better fatigue properties than those cut in the TD, because in the RD specimens more favorable crystallographic orientation is formed, which results in fewer slip systems activated during cyclic loading. Additionally, RD specimens typically exhibit a higher degree of grain elongation, which can promote a more homogenous deformation response during cyclic loading, further improving the fatigue life. In contrast, TD specimens tend to have a more heterogeneous deformation response during cyclic loading. This can result in a greater likelihood of crack initiation and propagation, leading to a reduced fatigue life.

Comparing the fatigue life results in a tensile test strain–stress diagram where the longer fatigue life is correlated with higher ultimate tensile strength (UTS) and smaller elongation at fracture. 

**Figure 6:**
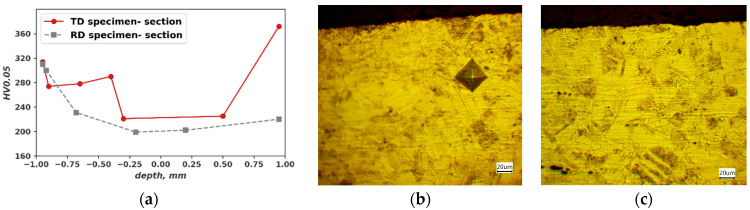
Cross-section close to broken surface of specimen TD (*N_f_* = 3.94 × 10^5^) and RD (*N_f_ =* 3.94 × 10^5^): (**a**) Microhardness profile; (**b**) Microstructure TD specimen; (**c**) Microstructure RD specimen.

The microhardness HV0.05 values measured in the cross-sections before and after the fatigue test are also compared. Before the fatigue test there are no significant differences in microhardness compared to the measure on a sheet surface 199 HV10. After the fatigue test higher microhardness values are observed close to the surface (Figure 6a). The main factor seems to be strain-induced martensite developed during cycling loading (Figure 6b,c).

### 3.2. Fatigue Life after Ball Burnishing 

Like non-burnished experiment, two groups of specimens according to sheet rolling direction are prepared. BB is conducted on both sides of the specimen’s high-stress area (Figure 2a), with burnishing regimes according to the experimental plan (Table 3). Some of the regimes are replicated. Fatigue testing is performed with the same loading as raw specimens. Results for the fatigue life expressed as cycles to failure are shown in Table 6. 

**Figure 7:**
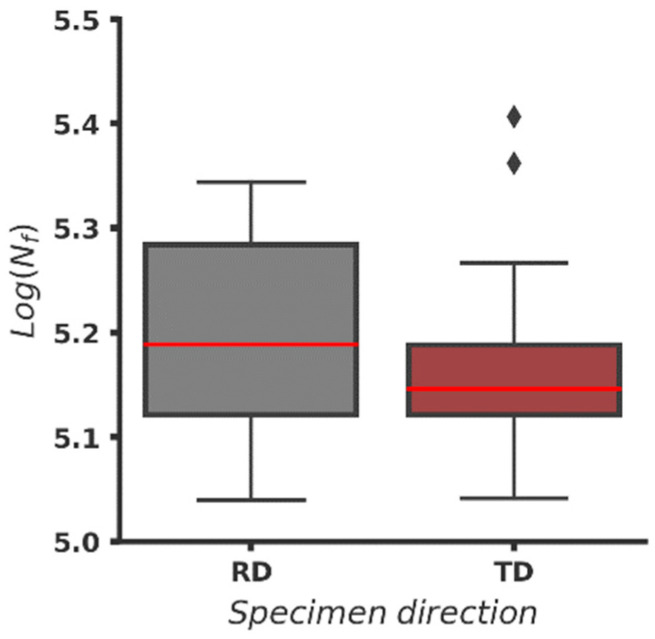
Fatigue life (*Log(N_f,burn_*)) of the BB specimens grouped by direction (Supplementary S1).

In Figure 7, overall fatigue data from Table 6, grouped by direction, is plotted. It is seen that boxes, which contain 50% of the data, overlap. The median value of fatigue life data from specimens cut in RD stays higher than the other median and is at the same level with the end of the box value of the fatigue life of specimens cut in TD, which is the indicator that it is likely that the fatigue life of RD specimens after BB is higher. It is expected since the raw specimens cut in RD also exhibit longer fatigue life. (See Section 3.1). The lower cup of both plots is on a similar level, but both boxes are skewed upward indicating higher variance of the data values higher than the median. The two higher results for the fatigue life of specimens cut in TD are far from the upper cup and seem to be outliers. As these specimens are BB with the highest force and longer fatigue life is expected, this is an indicator that burnishing regime parameters are also significant factors influencing the fatigue life. 

**Figure 8:**
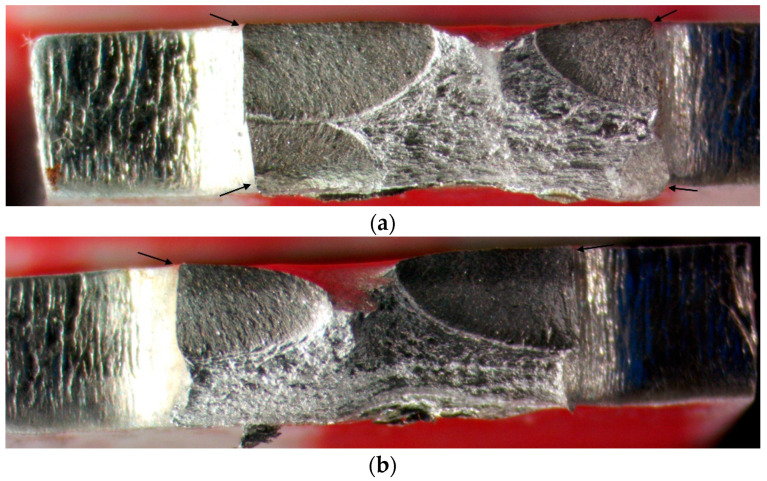
Broken surfaces of the BB with regime 5 (*F =* 1000 N, *f =* 300 mm/min) specimens: (**a**) RD (*N_f_ =* 1.54 × 10^5^); (**b**) TD (*N_f_ =* 1.34 × 10^5^).

Typical broken surfaces are given in Figure 8. Crack origin sites are the edges of the narrowest section. Cracks can initiate and propagate from any of the edges independently. Each crack propagates approximately to the middle of the section depth.

**Figure 9:**
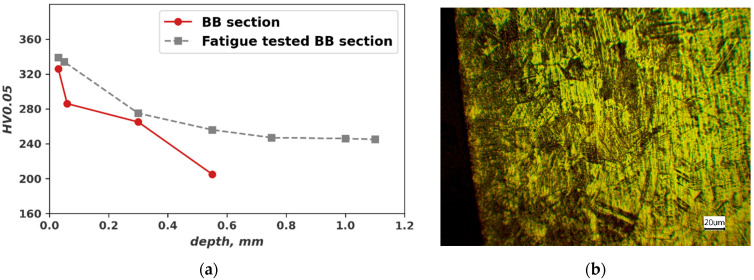
Cross-section of the BB with regime 5 (*F* = 1000 N, *f* = 300 mm/min) specimen TD (*N_f_* = 1.34 *×* 10^5^): (**a**) Microhardness profile before BB and after the fatigue test; (**b**) Microstructure after the fatigue testing.

Microhardness indentation traces are measured on a cross-section after burnishing and after the fatigue failure (Figure 9a). As for the non-burnished specimens, an increase in the hardness seems to be due to martensite formation during the cycling loading (Figure 9b). 

### 3.3. Gain of the Fatigue Life Due to Ball Burnishing

BB technology increases the fatigue life. The gain can be expressed in log scale as a ratio of the number of cycles to fatigue failure of BB and non-burnished specimens.

(7) $$G_{fl}=\log_{10}\left(\frac{N_{f,burn}}{N_{f}}\right)=\log_{10}\left(N_{f,\;burn}\right)-\log_{10}\left(N_{f}\right),$$

where $G_{fl}$ is the gain of fatigue life due to BB and $N_{f,burn},\;N_{f}$ are the number of cycles to failure of BB and non-burnished specimens, respectively. To get more physical meaning, the gain can be expressed in dB multiplying the *G_fl_* by 20. 

Due to the destructive nature of fatigue, it is impossible to test the same specimen twice (raw and BB). The simplest approach for obtaining the *N_f_* value is to take the mean result of tested raw specimens, but this approach will only scale the results from the BB specimens, not considering the uncertainty due to material local heterogeneity or/and specimen preparation. Here the posterior predictive distributions of the fatigue life of raw specimens ($N_{f}$), inferred from the probabilistic model explained in Section 3.1, are used to generate the batch of raw (non-BB) specimens. The algorithm to obtain the $\log_{10}\left(N_{f}\right)$ values is:

- Choose random values from posterior distributions for mean, standard deviation, and degree of freedom ($\mu_{i}|data;\;\sigma_{i}\left|data;\;\nu \right|data$);
- Generates random $\log_{10}\left(N_{f}\right)\;$ values from t-distribution with chosen values for $\mu_{i},\;\sigma_{i},\;\nu$.

**Figure 10:**
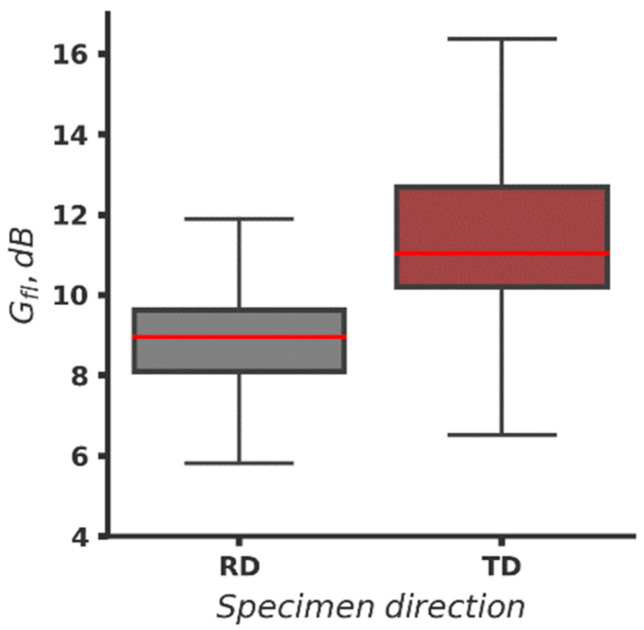
Gain of fatigue life (*G_fl_*, dB) due to BB of the specimens grouped by specimen cut direction.

Following the algorithm, 1000 rows with nine values each are generated for both kinds of specimens (RD and TD). Generated $\log_{10}\left(N_{f}\right)$ and experimental $\log_{10}\left(N_{f,\;burn}\right)$ values are sorted, assuming that specimens with longer fatigue life without BB will exhibit longer fatigue life after BB. The results for *G_fl_* in dB, grouped by the direction of the specimen cut, are summarized in Figure 10. To clarify the plot, outliers are removed. 

The gain of the fatigue life due to BB is in the range of approximately 6–12 dB with a median value around 9 dB for specimens cut in RD and in the range of 6.3–16.3 dB with a median of about 11 dB for the other group of specimens. The boxes do not overlap, so the probability for significant difference between both groups of data is high.

**Figure 11:**
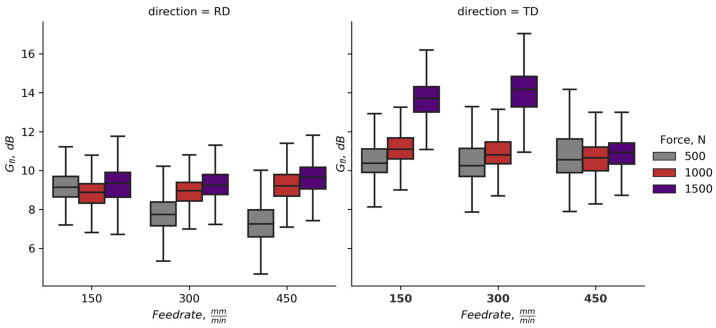
Gain of fatigue life (*G_fl_*, dB) due to BB of the specimens grouped by burnishing regime parameters (Supplementary S1).

In Figure 11, the data for gain of the fatigue life *G_fl_* grouped by the burnishing regime is shown. For all combinations of regime parameters, excluding the maximum force, feed-rate combination (1500 N, 450 mm/min), there is high probability for higher fatigue life gain of specimens cut in TD. The influence of the feed-rate is pronounced for the specimens cut in RD burnished with lower force (500 N), where the *G_fl_* decreases with the increase of the feed-rate. 

## 4. Discussion

Sakin [46] studies the mechanical properties and fatigue life of pure aluminum AA1050 and AA1100 sheets at room temperature. He prepared specimens selected in rolling and transverse directions and used a cantilever beam bending fatigue test at the same parameters of cyclic loading. The result from the experiments shows slightly higher strength (YS, UTS, and Bending strength) and longer fatigue life, especially in low cycle fatigue region, for specimens cut in the rolling direction. As the aluminum and austenite, which is a primary phase in AISI 304 steel, have the same type of face-centered cubic (FCC) lattice the results are like our findings. The microstructures are similar to one-phase grains with the exception of strain-induced martensite needles developed in some of the austenitic grains. Different direction behavior of rolled Mg-alloy AZ31 is reported in [47]. The main phase composition of this alloy has a hexagonal close-packed lattice. It shows strong anisotropic mechanical properties after rolling, but authors (F. Lv et al.) reported that the strength properties in tension (YS and UTS) in the TD are better than the in the RD. In tensile specimens selected in TD higher elongation was also registered which relates to fracture types. Specimens in the RD have fractures that show shearing, while the TD specimens show necking [47]. In strain-controlled and stress-controlled fatigue experiments, the fatigue life is longer for TD specimens, which correlates with their better strength properties. 

The results obtained in Section 3.3 for the fatigue life gain can be related to the peculiarities of the BB technology used. Since the BB tool makes additional oscillatory movement, the main deformation direction is normal to the main movement of the BB tool. The direction of predominant plastic deformation coincides with the direction in which the specimen is cut (Figure 4c,d). Therefore, as maximum strain in tension is higher in TD the improvement due to additional surface plastic deformation in the same direction is expected to be higher. 

Similar results for the mechanical properties of 0.8 mm rolled sheets from AISI 304 steel are obtained in [48]. The YS and UTS of RD- and TD-cut specimens are similar, but specimens cut in TD have higher elongation. The hardness of specimens before and after tensile testing is measured in three planes alongside the long side I, cross-section II, and top surface III. For the RD specimen’s hardness, improvement is lower in plane I than in the other two planes. In the TD specimens, the hardness increase is relatively balanced in all planes and slightly higher than the hardness of RD-cut specimens, showing the higher potential of strain hardening due to loading in TD. Of course, here, the hardness increase is due to strain hardening and the strain-induced martensite transformation should also be considered. The formation of strain induced α′-martensite during tensile testing is registered using XRD analysis and magnetic permeability change. The BB process also supports the martensitic transformation but mostly in the surface layer [49]. As the strain-induced transformation is sensitive to both strain and the strain rate [50] the burnishing regime parameters affect the fatigue life. In addition, there is a possibility of martensitic transformation during cycling loading [26], which is more pronounced for the prestrained specimens. All this supports the suggestion about optimal martensitic content for longer fatigue life [29]. 

## 5. Conclusions

The present study uses the vibration resonance bending fatigue test of 2 mm thick rolled sheet of SS 304 steel to compare the fatigue life of raw and BB specimens considering the direction of the specimen’s cut. A novel approach was used to obtain the complex toolpath of the burnishing tool, which has optimized length, calculated using a specially developed algorithm, which is based on the Euclidean distances between the toolpath’s points. As a result, the unfolded length of the toolpath was shortened by 5.7%, while the pattern of the cells’ distribution within the area of the regular relief, formed using the BB process, remains unchanged This may not look like a substantive toolpath optimization for the section with dimensions of 10 × 10 mm, but with larger sizes of the burnished surface, or if there are many sections that must be burnished, the overall cycle time reduction can be significant.

Results from fatigue life research show that the rolling direction should be considered when BB of thin rolled sheets is conducted. Specimens cut in RD have a longer fatigue life in correlation with slightly higher strength properties and lower elongation registered using the tensile test. If the predominant direction of BB deformation coincides with the direction in which the specimen is cut, longer fatigue life is likely to be obtained in specimens cut in RD. 

The probability of reaching the better gain of the fatigue life *G_fl_* is higher for the specimens cut in the transverse direction (TD) since the strain-hardening potential is higher. The higher gain of the fatigue life *G_fl_* > 10 dB is registered in TD-cut specimens BB with maximal force and a minimal or intermediate feed-rate. BB with predominant deformation direction normal to rolling direction can also be considered. 

According to burnishing regimes parameters, further microstructural analysis, and hardness and microhardness measurements of raw, BB, and fatigue-tested specimens are needed to clarify and obtain material-dependent fatigue life correlations. This will be the focus of our future work. 

## Figures and Tables

**Table 1:** Mechanical properties of austenitic stainless-steel 304 cold-rolled sheet.

		Mechanical Properties			
Material	Direction	Yield Strength, MPa	Ultimate Tensile Strength, MPa	Elongation A, %	HV10
304	RD	372 ± 10	710 ± 12	31.2 ± 0.8	199 ± 12
	TD	366 ± 8	678 ± 9	35.1 ± 0.6	

**Table 2:** Natural frequency and mode shape of cantilever beam with concentrated tip mass [38].

Boundary Conditions	Natural Frequency, *f*_1_ [Hz]	$\mathrm{Mode}\;\mathrm{Shape}\;\tilde{y}\left(x\right)$
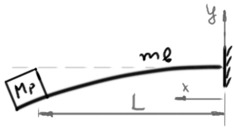	$\frac{1}{2\pi }\sqrt{\frac{3EI}{L^{3}.M_{eq}}}$ $M_{eq}=M_{p}+0.24m_{b}$	$\tilde{y}\left(x\right)={\left(\frac{x}{L}\right)}^{2}\left(3-\frac{x}{L}\right)$, for $0\leq x\leq \frac{L}{2}$

where *E =* 190 GPa is Young’s modulus of the SS 304; I = bh^3^/12 = 6.67 mm^4^–section moment of inertia; *M_eq_* = 36 g–equivalent tip mass.

**Table 3:** Experimental design and results for cycles to fatigue failure for RD and TD specimens.

Regime No	Coded Factors		Factors in Natural Values	
	A	B	Deforming Force, *F, N*	Feed-Rate, *f,* mm/min
1	−1	−1	500	150
2	−1	0	500	300
3	−1	+1	500	450
4	0	−1	1000	150
5	0	0	1000	300
6	0	+1	1000	450
7	+1	−1	1500	150
8	+1	0	1500	300
9	+1	+1	1500	450

**Table 4:** Cycles to fatigue failure for non-BB specimens selected in RD and TD directions.

No	Direction	*N_f_* × 10^4^	Log_10_ (*N_f_*)	Log_10_ (*N_f_*)_norm
**1**	RD	5.92	4.772	0.654
**2**	RD	6.71	4.827	1.290
**3**	RD	4.94	4.694	−0.264
**4**	RD	6.08	4.784	0.788
**5**	RD	4.96	4.695	−0.251
**6**	RD	5.51	4.741	0.289
**7**	TD	3.95	4.596	−1.412
**8**	TD	3.93	4.595	−1.430
**9**	TD	4.54	4.657	−0.699
**10**	TD	4.73	4.675	−0.485

**Table 5:** Summary of the Bayesian model for the fatigue life of raw specimens cut in RD and TD (Supplementary S1).

	Mean	Sd	HDI 2.5%	HDI 97.5%
$\mu_{RD}$	0.418	0.379	−0.344	1.191
$\mu_{TD}$	−1.015	0.480	−2.025	−0.043
$\sigma_{RD}$	0.824	0.382	0.292	1.602
$\sigma_{TD}$	0.837	0.507	0.220	1.979
ν	32.962	29.786	0.020	92.087

**Table 6:** Cycles to failure for BB specimens *N_f_ ×* 10^5^/Log_10_ *(N_f_).*

Direction	Replication	Burnishing Regime								
		1	2	3	4	5	6	7	8	9
RD	1	1.54/*5.188*	1.22/*5.085*	1.10/*5.040*	1.69/*5.228*	2.21/*5.349*	1.50/*5.177*	1.95/*5.291*	1.92/*5.284*	1.93/*5.284*
	2					1.32/*5.121*			1.23/*5.089*	
	3					1.54/*5.188*			1.77/*5.248*	
	Log_10_ *(N_f_) Mean*	*5.188*	*5.085*	*5.040*	*5.228*	*5.219*	*5.177*	*5.291*	*5.207*	*5.284*
	*St Dev*	*-*	*-*	*-*	*-*	*0.117*	*-*	*-*	*0.104*	*-*
TD	1	1.31/*5.117*	1.10/*5.042*	1.22/*5.087*	1.51/*5.178*	1.34/*5.126*	1.56/*5.193*	2.30/*5.362*	2.55/*5.407*	1.36/*5.133*
	2		1.29/*5.112*			1.53/*5.183*				1.85/*5.266*
	3		1.34/*5.127*			1.40/*5.146*				1.51/*5.178*
	Log_10_ *(N_f_) Mean*	*5.117*	*5.094*	*5.087*	*5.178*	*5.152*	*5.193*	*5.362*	*5.407*	*5.192*
	*St Dev*	*-*	*0.045*	*-*	*-*	*0.029*				*0.068*

## Supplementary Materials

The raw data from the fatigue experiments and Bayesian probabilistic model can be download from the GitHub repository of Diyan Dimitrov. Supplementary S1. Bayesian models https://github.com/DMDimitrovJ/304_Burnishing_Fatigue, accessed 5 May 2023.
